# Development and validation of an interpretable machine learning model for predicting postoperative fever after flexible ureteroscopic lithotripsy: a single-center retrospective cohort study

**DOI:** 10.3389/fmed.2026.1913792

**Published:** 2026-09-03

**Authors:** Xiaofei Lu, Chunping Yu, Zhiyong Ding, Sheng Luo, Xiaopeng Hu

**Affiliations:** 1 Beijing Chaoyang Hospital, Capital Medical University, Beijing, China; 2 Department of Urology, Xiangyang No.1 People’s Hospital Affiliated Hospital of Hubei University of Medicine, Xiangyang, China

**Keywords:** flexible ureterorenoscopy, kidney stones, machine learning, postoperative fever, SHapley Additive exPlanations

## Abstract

**Background:**

This study aimed to develop and validate an interpretable machine learning model to predict postoperative fever (POF) after flexible ureteroscopic lithotripsy (fURL).

**Objective:**

This study adopted a single-center retrospective temporal cohort design. Strictly adhering to predefined inclusion and exclusion criteria, patients with upper urinary tract stones (maximum diameter < 3 cm) who underwent flexible ureteroscopic lithotripsy (fURL) from January 2019 to December 2024 were enrolled as the derivation cohort, while consecutive cases treated between January and December 2025 were recruited to construct an independent temporal validation cohort. Least absolute shrinkage and selection operator (LASSO) regression filtered optimal predictors for postoperative fever (POF) risk modeling. Six machine learning (ML) algorithms included logistic regression (LR), random forest (RF), multi-layer perceptron (MLP), support vector machine (SVM), XGBoost, and LightGBM were built using combined clinical and imaging features to predict fURL-related POF. Model performance was assessed via multiple metrics including AUC and F1-score. The SHAP algorithm was employed to perform multi-dimensional interpretability analysis on the optimal model, thereby quantifying the contribution magnitude and predictive mechanism of each feature. Furthermore, the temporal validation cohort was used to evaluate the generalizability and extrapolation stability of the established model.

**Results:**

Among the six machine learning algorithms, The LightGBM model yielded the optimal discriminative metrics in this model comparison, including the maximum AUC (0.896, 95% CI: 0.853–0.940) and PR-AUC (0.787), as well as the highest specificity (0.967) and accuracy (0.891). DeLong pairwise comparisons demonstrated that LightGBM was statistically superior to four competing models; however, the AUC difference between LightGBM and XGBoost did not reach statistical significance. The SHAP analysis further clarifies the contribution of each variable in the prediction process. Temporal validation revealed acceptable temporal generalization performance of the LightGBM predictive model, with an AUC of 0.723 (95% CI: 0.648–0.799).

**Conclusion:**

This study employed an interpretable machine learning model to effectively predict POF following fURL. This model provides a reference for surgical risk stratification, which helps establish personalized postoperative monitoring strategies.

## Background

According to Global Burden of Disease Study 2021, there were approximately 106 million new global urolithiasis cases in 2021; the absolute disease burden rose due to population growth and aging with obvious gender, regional and socioeconomic disparities, while China bore nearly one-seventh of the global cases and presented an aging trend of urolithiasis patients (1). For upper urinary tract stones smaller than 2 cm, flexible ureteroscopic lithotripsy (fURL) has become a mainstream treatment, offering benefits such as minimal invasiveness, rapid recovery, and a lower risk of complications (2, 3).

Technological innovations in recent years, such as the integration of fURL with ureteral access sheath (UAS), improved laser lithotripsy platforms, and the advent of single-use flexible ureteroscopes, have markedly accelerated the clinical adoption of this technique across the globe, and some clinicians have even attempted to apply fURL for the management of renal stones larger than 3 cm (4–7). However, despite these advancements, postoperative fever (POF) remains one of the most common major complications following fURL. It may evolve into severe infectious conditions such as sepsis, potentially necessitating intensive care unit admission and even leading to mortality (8). So accurate prediction of POF risk is essential for minimizing postoperative complications and improving patient outcomes. Several studies have investigated the key risk factors for POF following fURL (9, 10). For example, Akgül et al. (9) demonstrated that patients with obesity, prolonged operative time, elevated SII, and elevated platelet-to-lymphocyte ratio (PLR) were at higher risk of POF development.

Advancements in data science have enabled machine learning (ML) methods—such as Random Forest, XGBoost, and LightGBM—to show significant potential in early disease prediction, particularly for postoperative complications (11–15). For example, ML has been used to predict stone-free rate (11), kidney stone composition (12), stone recurrence (13),sepsis (14),postoperative bleeding after percutaneous nephrolithotomy (15), among others. However, few studies have focused on the early prediction of postoperative fever after fURL. Machine learning models achieve high predictive accuracy yet remain well-known as “black boxes” with limited interpretability. Existing interpretability algorithms including Gradient SHapley Additive exPlanations (SHAP) frequently conflict with clinical experience, requiring further optimization and real-world clinical validation. Feature-based interpretation methods can effectively alleviate this black-box limitation (16).

This retrospective cohort study aimed to construct and validate an interpretable machine learning model for predicting POF following fURL. SHAP was adopted to enhance model interpretability and visualize the predictive contribution of individual variables.

## Patients and methods

### Study population

This retrospective single-center study included two independent cohorts of patients with upper urinary tract stones < 3 cm who received flexible ureteroscopic lithotripsy (fURL). Data from consecutive fURL patients treated between January 2019 and December 2024 formed the derivation cohort for preprocessing, model construction and internal validation. An additional temporal cohort of patients undergoing fURL between January 2025 and December 2025 was recruited to carry out temporal validation and evaluate the generalizability of the optimal model.

The inclusion criteria were as follows: (a) age 18–70 years; (b) diagnosis of upper urinary tract stones < 30 mm confirmed by computed tomography (CT); (c) availability of complete clinical data; and (d) surgery performed by urologists with > 5 years of endourological experience. Exclusion criteria included: (a) bilateral ureteral stones or solitary kidney; (b) impacted stones; and (c) coagulation disorders, abnormal urinary tract anatomy, or advanced malignant tumors.

### Ethical statements

This study protocol was reviewed and approved by the Biomedical Ethics Committee of Xiangyang No. 1 People’s Hospital, Affiliated Hospital of Hubei University of Medicine (MR-42-26-025583). All patient data were fully anonymized prior to analysis, and informed consent was waived given the retrospective nature of this study.

### Data collection and preprocessing

Data on patients with upper urinary tract stones were retrospectively extracted from the electronic medical record system, including demographic information, routine blood parameters, serum biochemical indicators, stone-related characteristics, and routine urinalysis profiles. To minimize potential selection bias, variables with a missing value rate greater than 20% were excluded from the analysis. Collinearity analysis was performed using the variance inflation factor (VIF) derived from multiple linear regression to evaluate correlations among variables. Variables with significant multicollinearity (VIF > 5) were screened, and only one indicator from each highly correlated pair was retained for subsequent feature selection to eliminate multicollinearity. The elimination priority was determined based on the clinical importance of the variables.

A total of 26 variables were ultimately included: Baseline basic information included gender, age, comorbidities (hypertension and diabetes), preoperative fever, application of the UAS device (all patients in our study received conventional ureteral access sheath, without suction-assisted S-UAS), and preoperative stent placement. Urinalysis included urine white blood cell, urine leukocyte esterase, urine nitrite, and midstream urine culture. Blood routine and blood biochemistry included Blood routine and serum biochemical parameters included white blood cell (WBC), platelet, red blood cell (RBC), hemoglobin (HB), serum creatinine (SCr), total bilirubin, albumin/globulin ratio (A/G), neutrophil-to-lymphocyte ratio (NLR), platelet-to-lymphocyte ratio (PLR), and lymphocyte-to-monocyte ratio (LMR). Stone-related characteristics included stone laterality, multiple stones, stone density, and the presence of hydronephrosis. Hydronephrosis was graded using the modified SFU classification: Grade 1–2 indicated mild-to-moderate pelvicalyceal dilation, while Grade 3–4 represented severe dilation accompanied by renal parenchymal thinning.

### Definitions of POF

Postoperative fever was defined as a body temperature exceeding 38 °C within 72 h after surgery, and in the absence of signs of infection in other organ systems (9). Prophylactic antibiotics, mainly cephalosporins or penicillins, were administered 30 min preoperatively. A positive midstream urine culture was defined as pathogenic bacterial colony counts ≥ 10^5^ CFU/mL; all patients underwent routine midstream urine culture testing within 72 h after hospital admission. For patients with positive preoperative urine culture or bacteriuria, anti-infective therapy was given first, and surgery was performed only after urine culture results turned negative. In line with AUA and EAU guidelines, patients with ureteral calculi complicated by preoperative fever and urinary obstruction received urgent urinary decompression and broad-spectrum antibiotic treatment, and elective lithotripsy was arranged after infection resolution. The variable positive preoperative urine culture and preoperative fever in our model refers to initial admission baseline culture result. Preoperative fever was documented for any temperature ≥ 38.0 °C at any admission time point within 14 days before surgery.

### Feature selection

All patients who underwent fURL were randomly divided into the training cohort and validation cohort at a 7:3 ratio. In the training cohort, missing data were handled via multiple imputation by chained equations (MICE) with 10 imputation datasets and 20 iterative cycles to achieve convergence for continuous variables, and modal imputation was adopted for categorical variables to minimize the bias caused by missing values. The imputation model incorporated all demographic, laboratory, stone and surgical covariates, while the primary outcome (postoperative fever) was excluded from imputation predictors to eliminate data leakage. The least absolute shrinkage and selection operator (LASSO) regression was used for high-dimensional variable reduction and to screen predictors of postoperative fever after fURL. We performed ten-fold cross-validation to select the optimal lambda value, and lambda.min (the lambda corresponding to the minimum cross-validation error) was adopted for variable selection. Variables with non-zero regression coefficients were reserved as candidate predictors. Finally, candidate variables were further selected for the machine learning (ML) model based on LASSO results and comprehensive clinical judgment.

### Model development and comparison

To address the class imbalance characterized by abundant negative samples and limited positive cases, we adopted an oversampling technique to improve model predictive ability. Borderline SMOTE, an improved oversampling algorithm, was utilized to construct machine learning models in the training cohort.

Using the aforementioned variables, we constructed predictive models to forecast POF after fURL, employing six ML algorithms: logistic regression (LR), random forest (RF), multi-layer perceptron (MLP), support vector machine (SVM), Extreme Gradient Boosting (XGBoost), and Light Gradient Boosting Machine (LightGBM). To evaluate the reliability of these models, multiple common evaluation metrics were adopted, including AUC (area under the ROC curve), sensitivity, specificity, accuracy, and F1 score. Youden’s J index was further calculated to comprehensively assess the model’s ability to balance sensitivity and specificity. Finally, bootstrap resampling with 1,000 repetitions was conducted on the training cohort to perform internal validation and assess the stability of the prediction model.

To ensure transparency in model development, validation, and manuscript preparation, the full workflow of our predictive model was rigorously designed following the TRIPOD-AI reporting checklist (17). The PROBAST-AI tool was utilized to thoroughly assess the overall risk of bias across this single-center retrospective study (18). Moreover, conventional statistical methods including calibration plots and Decision Curve Analysis (DCA) were applied to assess model calibration and quantify the clinical net benefit of the predictive model (19, 20).

### Model interpretability

SHapley Additive exPlanations was adopted to interpret the established machine learning model. SHAP visualizations were generated to exhibit individualized feature importance.

### Temporal validation

We performed temporal validation to evaluate the generalizability of our final predictive model. The discriminative performance was quantified using the area under the receiver operating characteristic curve (AUC) with corresponding 95% confidence intervals (CIs). The optimal cut-off threshold was predefined by maximizing the Youden’s J statistic within the training subset of the derivation cohort; this fixed threshold was not re-calibrated or re-optimized in the temporal validation cohort. On the basis of this prespecified threshold, we additionally calculated complementary diagnostic metrics, including accuracy, sensitivity, specificity, and the F1-score.

### Statistical analysis

The Kolmogorov–Smirnov (K–S) test was used to assess the normality of continuous variables. Normally distributed continuous variables were compared using Student’s *t*-test and presented as mean ± standard deviation. Non-normally distributed continuous variables were analyzed by the Mann–Whitney U test and described as median with interquartile range. Categorical variables were compared using the chi-square test or Fisher’s exact test and reported as frequencies and percentages. All statistical tests were two-tailed, and a *p*-value < 0.05 was considered statistically significant.

All statistical and computational analyses were performed using R 4.6.1^1^ and Python 3.11.5.^2^ Software packages utilized in this study were as follows: R (packages: glmnet for LASSO regression, mice for multiple imputation of missing data, DMwR for SMOTE oversampling, pROC for ROC curve and AUC calculation), and Python (libraries: scikit-learn, LightGBM, XGBoost, and SHAP for model construction and post-hoc interpretability analysis).

## Results

### Patient characteristics

**FIGURE 1:**
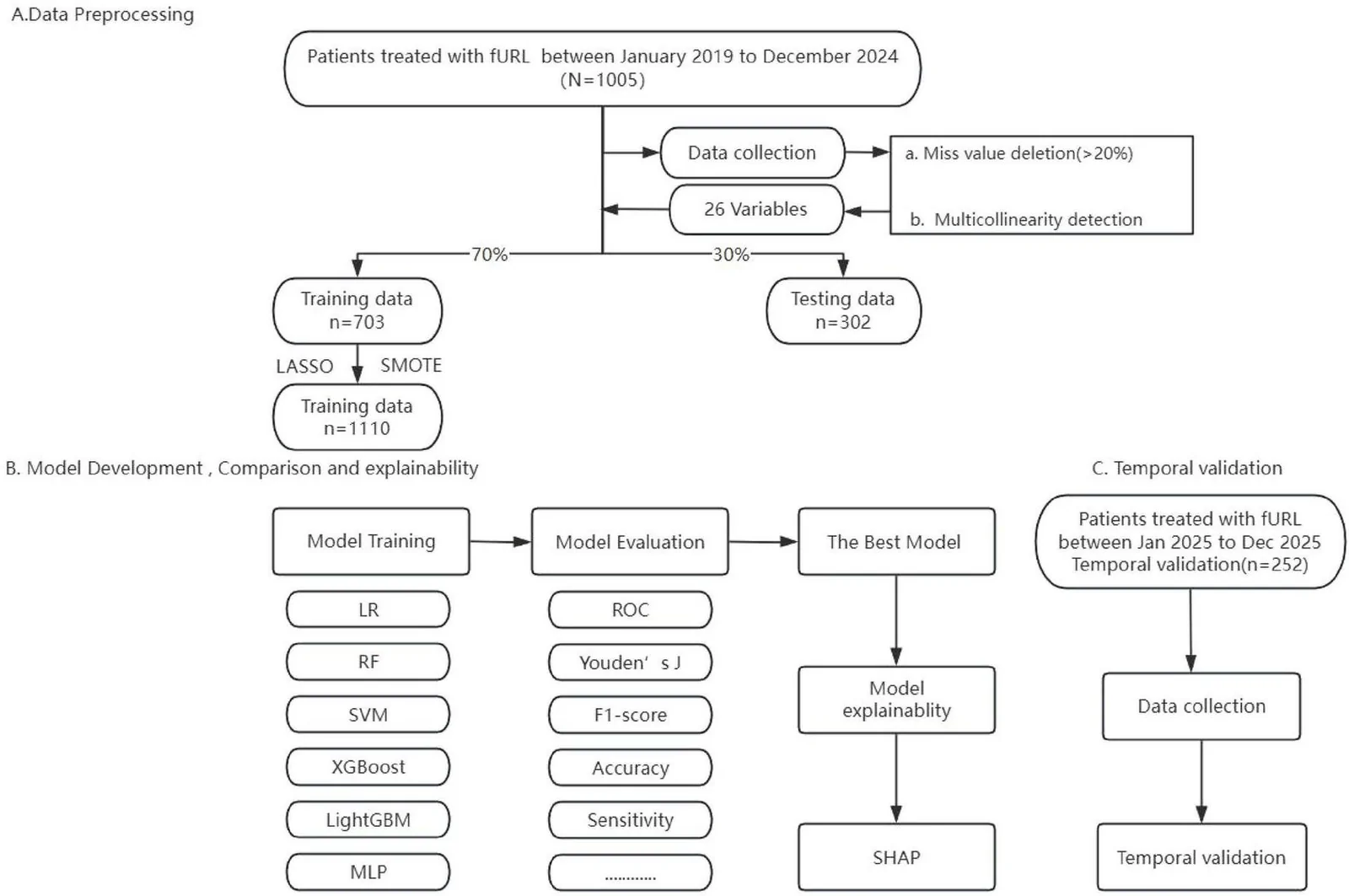
Flow chart of the study. **(A)** Data preprocessing workflow; **(B)** Model development, comparison and explainability analysis; **(C)** Temporal validation procedure.

Figure 1 illustrates the overall design procedure of the present study. A total of 1,005 patients undergoing flexible ureteroscopic lithotripsy were enrolled in this study for analysis of their demographic and clinical profiles. Postoperative fever developed in 211 cases, accounting for 20.9%. Table 1 summarizes the intergroup differences in demographic characteristics, routine blood tests, biochemical indices, preoperative imaging results and routine urine parameters. The median age was comparable between the two groups (both 52 [42, 60] years, *P* = 0.824). Significant between-group differences were observed in sex distribution (*P* = 0.017), with a higher proportion of females in the postoperative fever group (41.7% vs. 32.6%). No significant differences were detected in the prevalence of hypertension (*P* = 0.464), diabetes (*P* = 0.212), or preoperative stenting (*P* = 0.372). Patients who developed postoperative fever after fURL had a significantly higher rate of preoperative fever (39.8% vs. 4.2%, *P* < 0.001) and a significantly lower rate of UAS usage (29.5% vs. 19.9%, *P* = 0.003).

Urine tests also differed statistically between the two groups. The postoperative fever group presented a significantly higher mean urine white blood cell (WBC) count (556.88 ± 3,119.73 vs. 186.68 ± 751.21, *P* = 0.002), a greater proportion of grade 3+ urine leukocyte esterase (28.9% vs. 11.5%, *P* < 0.001), a higher positive rate of urine nitrite (10.4% vs. 5.5%, *P* = 0.017), and an elevated prevalence of positive midstream urine culture (32.2% vs. 17.9%, *P* < 0.001).

In terms of blood routine and biochemical indicators, patients who developed postoperative fever after fURL had a significantly higher median WBC (*P* < 0.001), red blood cell count (*P* = 0.048), platelet count (*P* = 0.017), and neutrophil-lymphocyte ratio (NLR) (*P* = 0.003), as well as lower hemoglobin (HB) (*P* = 0.002), lymphocyte-monocyte ratio (LMR) (*P* = 0.035), and albumin-globulin ratio (A/G) (*P* = 0.003). Whereas platelet-lymphocyte ratio (PLR), serum creatinine (SCr), and total bilirubin showed no significant differences (*P* > 0.05) between the two group.

Regarding radiological features, patients who developed postoperative fever after fURL more frequently had multiple stones (47.9% vs. 39.5%, *P* = 0.035) and severe hydronephrosis (Grade 3–4, 35.1% vs. 25.8%, *P* = 0.028).

**TABLE 1:** Baseline characteristics of the study group.

Variables	Post.op.fever (−)	Post.op.fever (+)	*P*-value*
Age, years median [IQR]	52 [42, 60]	52 [42, 60]	0.824
**Sex, *n* (%)**			0.017*
Female	259 (32.6)	88 (41.7)	
Male	535 (67.4)	123 (58.3)	
**Hypertension, *n* (%)**			0.464
No	522 (65.7)	145 (68.7)	
Yes	272 (34.3)	66 (31.3)	
**Diabetes, *n* (%)**			0.212
No	708 (89.2)	181 (85.8)	
Yes	86 (10.8)	30 (14.2)	
**Pre-op-fever, *n* (%)**			< 0.001*
No	761 (95.8)	127 (60.2)	
Yes	33 (4.2)	84 (39.8)	
**UAS, *n* (%)**			0.003*
No	152 (19.9)	60 (29.5)	
Yes	642 (80.1)	151 (71.5)	
**Pre-stented, *n* (%)**			0.372
No	645 (81.2)	165 (78.2)	
Yes	149 (18.8)	46 (21.8)	
Urine.WBC, (/μL) median [IQR]	26 [5–851]	58 [24–1,205.5]	0.002*
**Urine.leukocyte.esterase, *n* (%)**			< 0.001*
0	525 (66.1)	106 (50.2)	
1+	102 (12.8)	26 (12.3)	
2+	76 (9.6)	18 (8.5)	
3+	91 (11.5)	61 (28.9)	
**Urine.nitrite, *n* (%)**			0.017*
No	750 (94.5)	189 (89.6)	
Yes	44 (5.5)	22 (10.4)	
**Mid.urine.culture, *n* (%)**			< 0.001*
No	652 (82.1)	143 (67.8)	
Yes	142 (17.9)	68 (32.2)	
WBC, (×10^9^/L) median [IQR]	6.44 [5.25, 8.18]	7.26 [5.79, 9.81]	< 0.001*
RBC, (×10^12^/L) median [IQR]	4.56 [4.22, 4.91]	4.46 [4.13, 4.88]	0.048
Platelet, (×10^9^/L) median [IQR]	220 [184.25, 262]	233 [190.5, 283.5]	0.017*
HB, (g/L) median [IQR]	140 [128.25, 150]	135 [125, 148]	0.002*
NLR, median [IQR]	2.60 [1.80, 3.99]	2.85 [1.96, 5.23]	0.003*
PLR, median [IQR]	141.36 [109.52, 191.84]	149.44 [114.51, 205.89]	0.061
LMR, median [IQR]	3.60 [2.57, 4.73]	3.29 [2.21, 4.62]	0.035*
SCr, (μmol/L) median [IQR]	78.45 [64.60, 95.97]	78.00 [61.75, 98.65]	0.703
Total.bilirubin, μmol/L median [IQR]	15.3 [11.6, 20]	14.2 [11.45, 18.55]	0.083
A/G, median [IQR]	1.6 [1.4, 1.8]	1.5 [1.35, 1.7]	0.003*
**Stone side, *n* (%)**			1.000
Left	418 (52.6%)	111 (52.6%)	
Right	376 (47.4%)	100 (47.4%)	
**Multiple stones, *n* (%)**			0.035*
No	480 (60.5%)	110 (52.1%)	
Yes	314 (39.5%)	101 (47.9%)	
Stone size, (mm) median [IQR]	10.00 [10.00, 12.07]	10.00 [10.00, 12.80]	0.711
Stone density, (HU) median [IQR]	800.00 [546.5, 1,049.25]	817.00 [600, 1,093]	0.131
**Hydronephrosis, *n* (%)**			0.028*
Grade 1–2	589 (74.2%)	137 (64.9%)	
Grade 3–4	205 (25.8%)	74 (35.1%)	

IQR, interquartile range; pre-op-fever, preoperative fever; UAS, ureteral access sheath; WBC, white blood cell; RBC, red blood cell; HB, hemoglobin; NLR, neutrophil-to-lymphocyte ratio; PLR, platelet-to-lymphocyte ratio; LMR, lymphocyte-to-monocyte ratio; SCr, serum creatinine; A/G, albumin-to-globulin; SD, standard deviation; HU, Hounsfield Units. *Statistically significant.

### Feature selection

**FIGURE 2:**
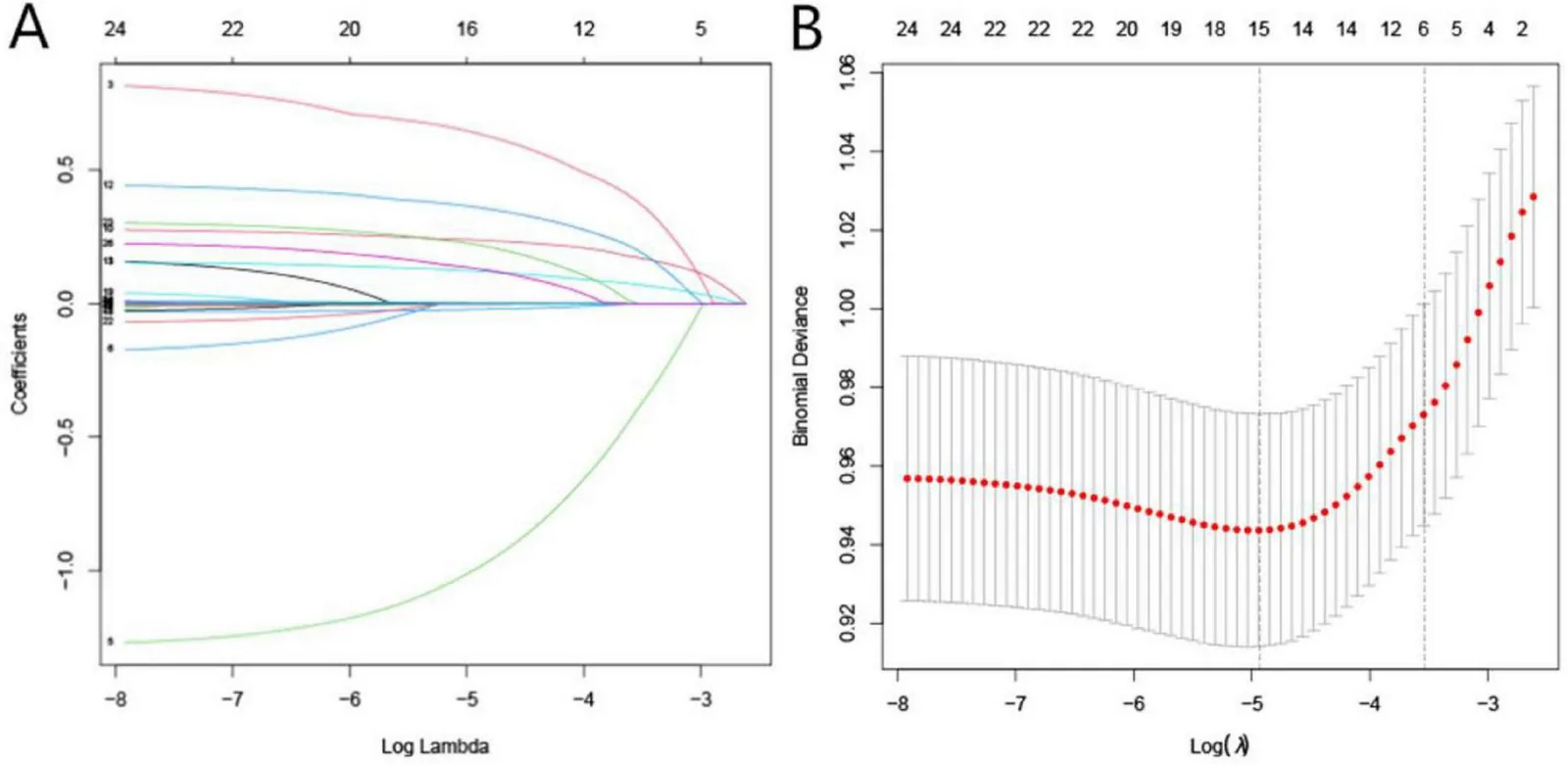
LASSO binary logistic regression was applied for the selection of radiomics and clinical features. **(A)** Optimal parameter (lambda) selection in the LASSO model was performed using ten-fold cross-validation based on the minimum criterion. **(B)** LASSO coefficient proffles of the 26 features.

Of demographic characteristics (including age and gender), preoperative complete blood count parameters, urinalysis parameters and radiological features, the LASSO binary logistic regression model was applied to reduce 26 features to 15 potential predictors (Figures 2A,B). Although age and sex had zero LASSO coefficients (weak linear association with POF), previous literature confirmed their nonlinear correlation with postoperative urinary infection risk; tree-based LightGBM can capture nonlinear interaction effects, so we retained them to avoid omitting clinically meaningful stone morphological predictors. We listed the full 15 LASSO-selected variables and two manually supplemented variables, we ultimately included 17 variables for subsequent machine learning analysis: age, gender, pre-op fever, UAS, urine WBC, urine leukocyte esterase, mid-urine culture, WBC, PLR, Platelet, HB, SCr, total bilirubin, multiple stones, stone size, hydronephrosis, and stone density.

### Evaluation of ML models

**FIGURE 3:**
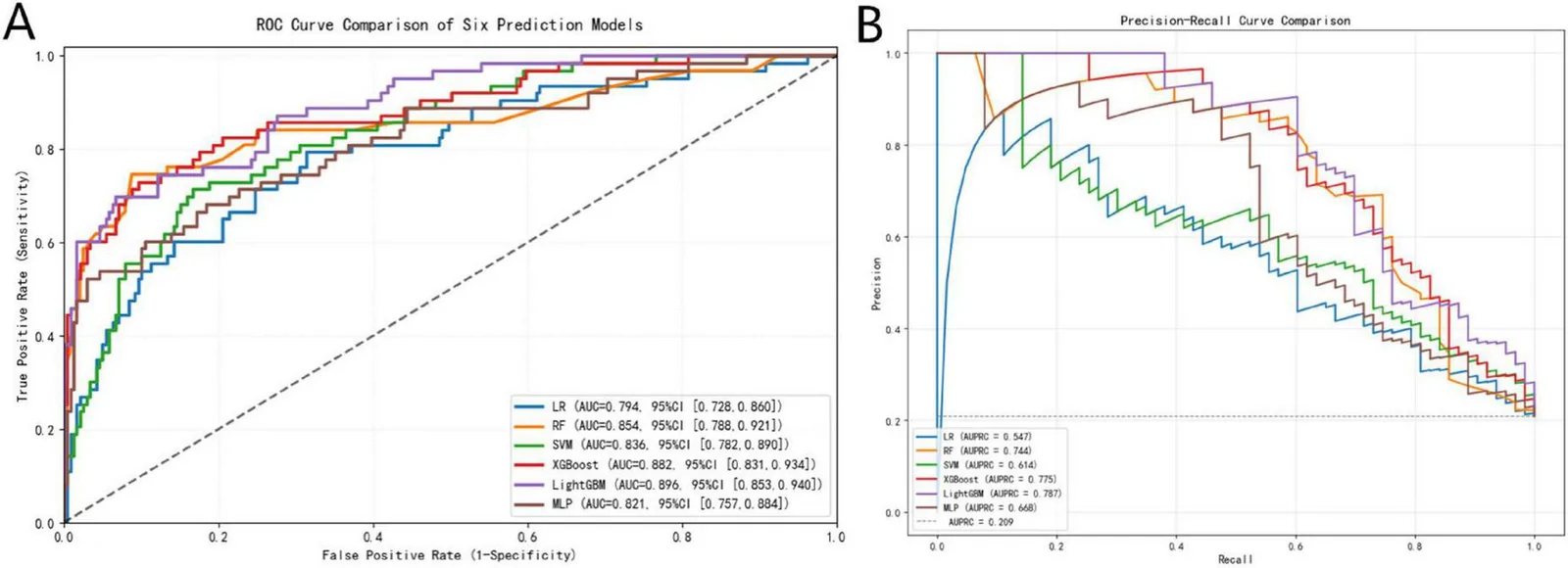
Predictive performance of six machine learning models for POF after fURL. **(A)** ROC curves; **(B)** PRC curves.

**FIGURE 4:**
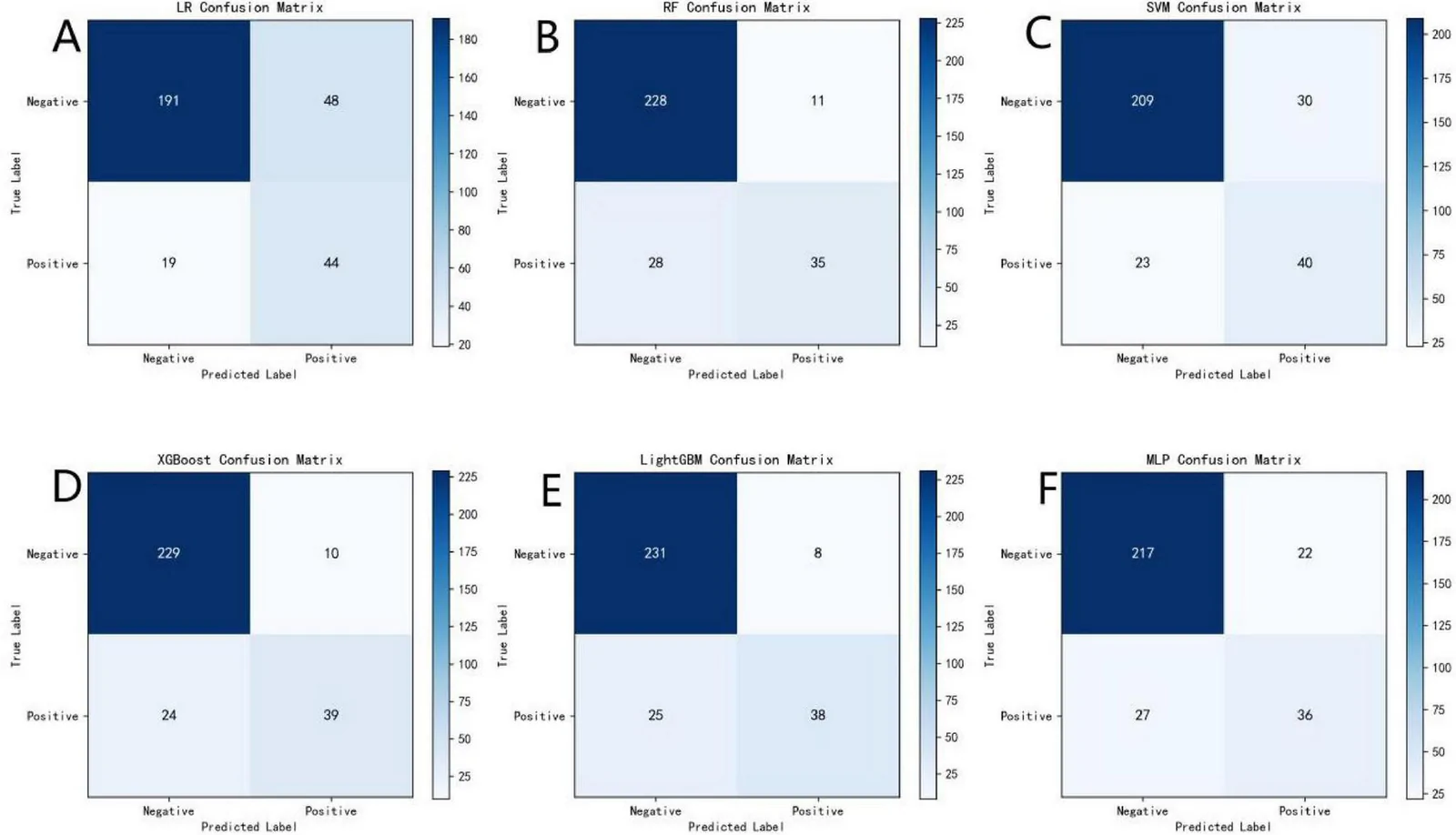
Confusion matrices of the six ML models. **(A)** Logistic regression; **(B)** random forest; **(C)** support vector machine; **(D)** XGBoost; **(E)** LightGBM; **(F)** multi-layer perceptron.

Six machine learning models were adopted in this study to predict postoperative fever after fURL, including LR, RF, MLP, SVM, XGBoost, and LightGBM. The ROC curves, precision-recall curve (PRC) curves, and confusion matrix of each model are presented in Figures 3A,B, 4A–F. Among the six ML models, LightGBM model had the highest AUC. Other metrics of the six ML models are shown in Table 2. The LightGBM model yielded the optimal discriminative metrics in this model comparison, with the highest AUC (0.896 95% CI: 0.853–0.94) and PR-AUC (0.787) among all models, and also achieved the highest specificity (0.967) and the highest accuracy (0.891), showing acceptable balance between missed diagnosis and misdiagnosis control,.

**TABLE 2:** Comparison of performance parameters among machine learning models.

Model	AUC (95%CI)	PR-AUC	Accuracy	Sensitivity	Specificity	F1-score	Youden’s J
LR	0.794 (0.728–0.86)	0.547	0.778	0.698	0.799	0.567	0.497
RF	0.854 (0.788–0.921)	0.744	0.871	0.555	0.954	0.642	0.509
SVM	0.836 (0.782–0.89)	0.614	0.824	0.634	0.874	0.601	0.509
XGBoost	0.882 (0.831–0.934)	0.775	0.887	0.619	0.958	0.696	0.577
LightGBM	0.896 (0.853–0.94)	0.787	0.891	0.603	0.966	0.697	0.569
MLP	0.821 (0.757–0.884)	0.668	0.837	0.571	0.908	0.595	0.479

AUC, area under the curve of ROC; PR-AUC, area under the precision-recall curve; LR, logistic regression; RF, random forest; SVM, support vector machine; XGBoost, eXtreme gradient boosting; LightGBM, Light Gradient Boosting Machine; MLP, multi-layer perceptron.

Table 3 presents pairwise statistical comparisons of the AUC values across all six predictive models using DeLong’s test to identify significant discrepancies in discriminative performance. LightGBM exhibited significantly superior discriminative performance relative to most competing models (all *P* < 0.05), with the largest statistical difference detected in the comparison with conventional logistic regression. Nevertheless, no statistically significant difference in AUC was identified between LightGBM and XGBoost, indicating that these two algorithms afforded comparable overall predictive discrimination.

**TABLE 3:** DeLong test results for AUC comparison.

Models	LR	RF	SVM	XGBoost	LightGBM	MLP
LR	1.0000	0.0409	0.1255	0.0213*	0.0013*	0.3429
RF	0.0409*	1.0000	0.3995	0.5205	0.0380*	0.3594
SVM	0.1255	0.3995	1.0000	0.1661	0.0088*	0.8156
XGBoost	0.0213*	0.5205	0.1661	1.0000	0.1089	0.1473
LightGBM	0.0013*	0.0380*	0.0088*	0.1089	1.0000	0.0114*
MLP	0.3429	0.3594	0.8156	0.1473	0.0114*	1.0000

*Statistically significant.

The model parameters are as follows: random_state = 42, learning rate = 0.1, number of leaves = 31, maximum tree depth = −1, minimum number of samples per leaf = 20, number of estimators or boosting rounds = 100, subsampling parameters = 1, feature fraction = 1, regularization parameters = L1: 0.0, L2: 0.0, early-stopping strategy = not used, hyperparameter optimization method = no, hyperparameter search space = not applied, random seed = 42, software packages and package versions = python: 3.11.5; pandas: 3.0.2; numpy: 1.26.4; scikit-learn: 1.8.0; lightgbm: 4.6.0; xgboost: 3.2.0; joblib: 1.5.3.

Then we plotted calibration curves and decision curve analysis (DCA) to assess the calibration and clinical net benefit of the LightGBM predictive model in the internal test set (Supplementary Figure 1). For calibration: the observed event rate (green line) closely matched the perfect calibration line at low-to-moderate predicted risks, with minor deviation only at high risk. The Brier score was 0.1022, reflecting low overall prediction error and acceptable calibration. For DCA : the LightGBM curve retained positive net benefit over a broad threshold range (0–0.80), superior to the “treat-all” and “treat-none” strategies. Net benefit turned negative above a threshold of 0.80, where model-guided intervention offered no clinical advantage.

### Model interpretability

**FIGURE 5:**
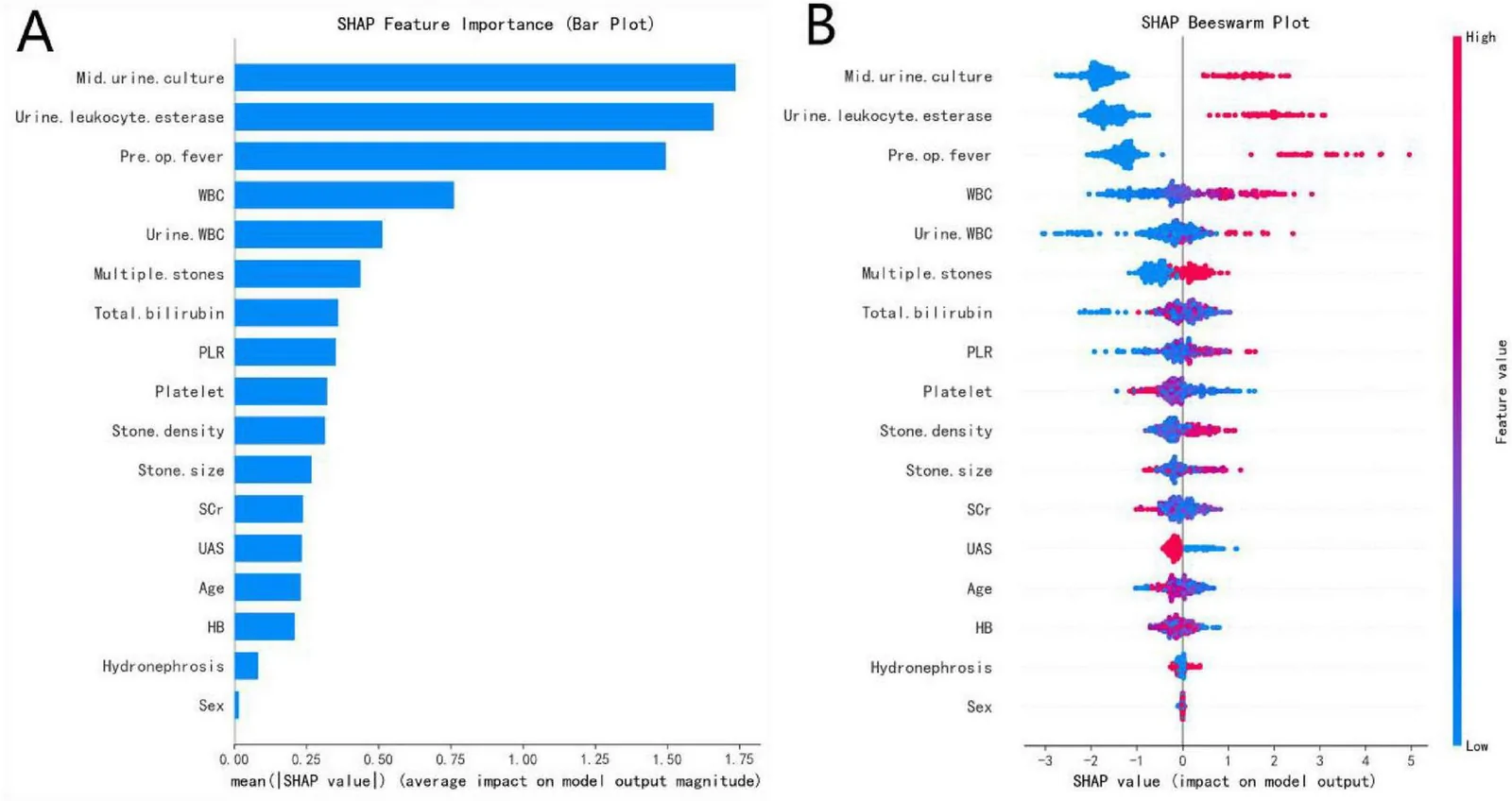
SHAP feature importance. **(A)** Bar chart of the all features; **(B)** Beeswarm plot of the all features.

Since the LightGBM model exhibited the optimal overall predictive efficacy among the six machine learning algorithms, it was selected for subsequent model interpretation analysis. The feature importance rankings derived from the LightGBM model are illustrated in Figures 5A,B. The top ten influential predictors in descending order were midstream urine culture, urine leukocyte esterase, preoperative fever, WBC, Urine WBC, multiple stones, total bilirubin, PLR, Platelet and stone density. Figure 5A shows the SHAP values of all features. The SHAP beeswarm plot in Figure 5B characterizes the SHAP value distribution of individual variables, visually depicting how each feature contributes to model predictions within different value intervals. A color gradient ranging from blue (lower feature values) to purple (higher feature values) is applied to quantify the correlation between feature magnitude and predictive performance. Among all variables, midstream urine culture exhibited the strongest SHAP contribution. Higher feature values (marked in pink) were associated with elevated SHAP values, indicates that a positive midstream urine culture exerts a substantial positive effect on the model’s risk prediction. Urine leukocyte esterase, preoperative fever, and multiple stones were also important predictive features; their positive status tended to increase the model’s predicted risk. Furthermore, other parameters such as WBC, age, SCr, total bilirubin and stone density also presented considerable impacts on the predictive performance of the model. In contrast, intraoperative application of UAS usage was associated with a reduced predicted risk derived from the model.

**FIGURE 6:**
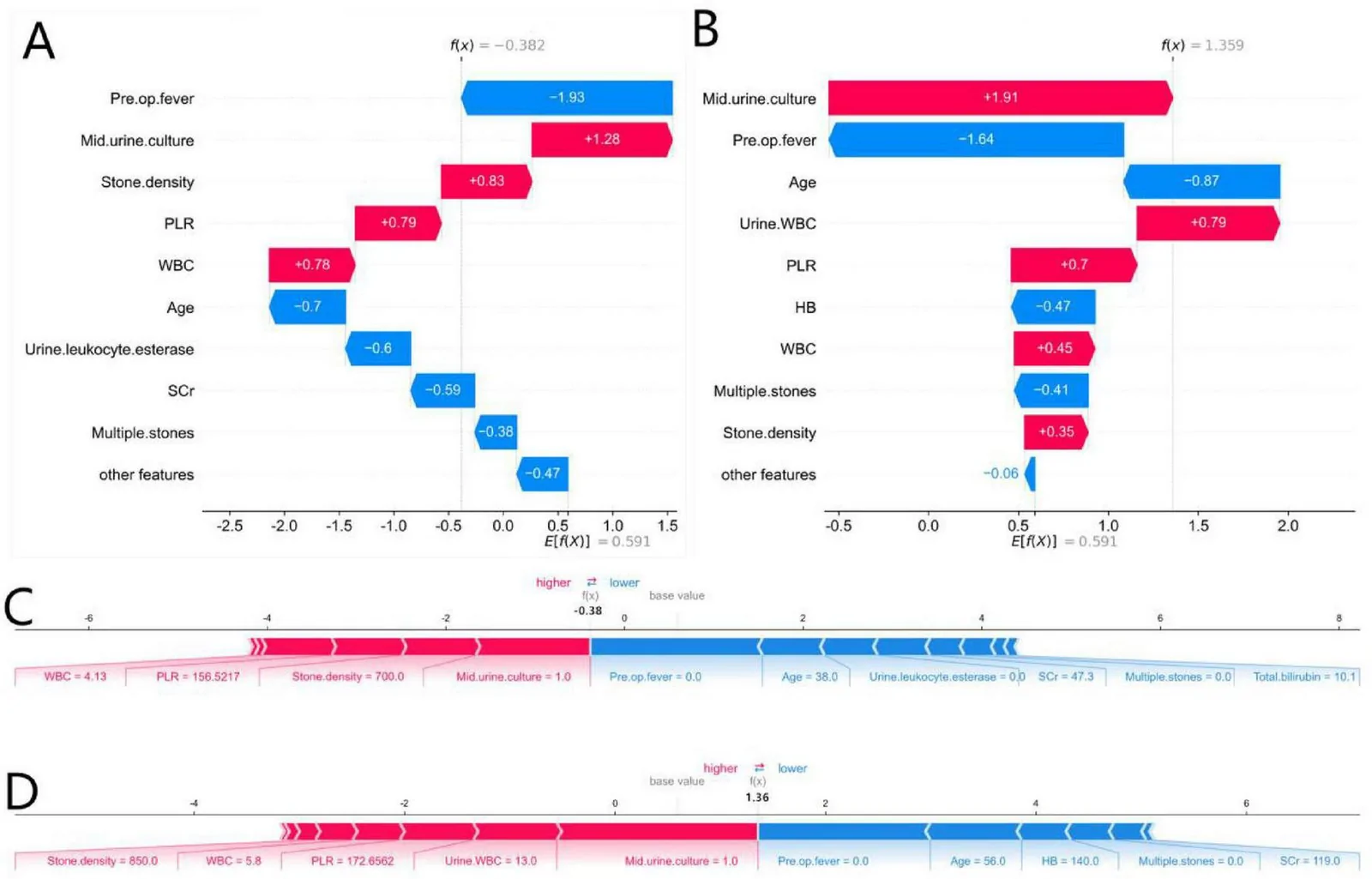
Local model explanation by the SHAP method. **(A)** Waterfall plot for one non-POF patient. **(B)** Waterfall plot for one POF patient. **(C)** Force plot for one non-POF patient. **(D)** Force plot for one POF patient.

Waterfall plots (Figures 6A,B) intuitively quantified individual feature contributions for typical patients, with blue bars representing risk-reducing negative effects and red bars representing risk-increasing positive effects. Force plots (Figures 6C,D) further demonstrated how each feature shifted predictions from the baseline base value to the final output, with blue and red arrows indicating protective and risk-increasing effects, respectively.

The same patient without postoperative fever is presented in both Figures 6A,C. For this individual, preoperative fever status, age, and urine leukocyte esterase level served as the predominant risk-mitigating factors, which drove a marked reduction in the model-predicted probability of postoperative fever, thus confirming the patient’s low risk profile. Conversely, midstream urine culture positivity and stone density were the primary risk-elevating factors contributing to postoperative fever risk in this patient. The model yielded a final prediction value of −0.382 for this patient, further confirming the patient’s low risk profile for developing postoperative fever, consistent with the aforementioned feature contribution analysis results. The same patient with postoperative fever is depicted in both Figures 6B,D. For this individual, a positive midstream urine culture (MSUC) result was the dominant factor driving a sharp rise in the model’s predictive risk score. The model ultimately generated a final prediction value of 1.359 for this patient, confirming an extremely high risk profile for developing postoperative fever, consistent with the risk factor contribution analysis presented earlier.

### Temporal validation

We conducted temporal validation to evaluate the generalizability of the LightGBM predictive model. Table 4 summarizes the intergroup differences in demographic characteristics, routine blood tests, biochemical indices, preoperative imaging results and routine urine parameters of the temporal validation dataset. Supplementary Table 1 shows the baseline characteristics of patients in the derivation and temporal validation cohorts. Statistically significant differences were only detected for stone laterality (*P* = 0.001) and urine leukocyte esterase grade (*P* < 0.001); all other variables showed comparable distributions (*P* > 0.05).

**TABLE 4:** Baseline characteristics of temporal validation dataset.

Variables	Post.op.fever (−)	Post.op.fever (+)	*P*-value*
Age, years mean ± SD	49.56 ± 12.02	52.96 ± 13.87	0.078
**Sex, *n* (%)**			0.049*
Female	70 (35.4)	27 (50)	
Male	128 (64.6)	27 (50)	
**Hypertension, *n* (%)**			0.835
No	129 (65.2)	36 (66.7)	
Yes	69 (34.8)	18 (33.3)	
**Diabetes, *n* (%)**			0.073
No	176 (88.9)	43 (79.6)	
Yes	22 (11.1)	11 (20.4)	
**Pre-op-fever, *n* (%)**			0.001*
No	184 (92.9)	42 (77.8)	
Yes	14 (7.1)	12 (22.2)	
**UAS, *n* (%)**			0.722
No	41 (20.7)	10 (18.5)	
Yes	157 (79.3)	44 (81.5)	
**Pre-stented, *n* (%)**			0.178
No	166 (83.8)	41 (75.9)	
Yes	32 (16.2)	13 (24.1)	
Urine.WBC, (/μL) median (IQR)	24 (7–86.5)	54 (13–265.7)	0.002*
**Urine.leukocyte.esterase, *n* (%)**			< 0.001*
0	160 (80.8)	32 (59.3)	
1+	28 (14.2)	15 (27.7)	
2+	5 (2.5)	4 (7.4)	
3+	5 (2.5)	3 (5.6)	
**Urine.nitrite, *n* (%)**			0.741
No	187 (94.4)	52 (96.3)	
Yes	11 (5.6)	2 (3.7)	
**Mid.urine.culture, *n* (%)**			< 0.001*
No	172 (86.9)	32 (59.3)	
Yes	26 (13.1)	22 (40.7)	
WBC, (×10^9^/L) median [IQR]	6.4 (5.2, 8.5)	8.1 (6–11.7)	< 0.001*
RBC, (×10^12^/L) mean ± SD	4.5 ± 0.6	4.6 ± 0.7	0.931
Platelet, (×10^9^/L) mean ± SD	225.04 ± 62.53	242.09 ± 79.72	0.096
HB, (g/L) mean ± SD	138.1 ± 16.3	134.1 ± 16.7	0.11
NLR, median [IQR]	2.8 [2.1, 5.1]	2.9 [1.9–5.6]	0.923
PLR, median [IQR]	154.4 [118.1, 197.6]	149.6 [116.2, 197.9]	0.994
LMR, median [IQR]	3.21 [2.31, 4.26]	3.14 [2.12, 4.97]	0.542
SCr, [μmol/L] median [IQR]	79.1 [64.6, 96.2]	77.5 [59.9, 102.5]	0.619
Total.bilirubin, μmol/L median [IQR]	15.8 [11.3, 19.9]	14.6 [11.7, 20.2]	0.626
A/G, median [IQR]	1.6 [1.5, 1.7]	1.7 [1.5–1.8]	0.232
**Stone side, *n* (%)**			0.032*
Left	87 (43.9)	15 (27.7)	
Right	111 (56.1)	39 (72.3)	
**Multiple.stones, *n* (%)**			0.842
No	113 (57.1)	30 (55.5)	
Yes	85 (42.9)	24 (45.5)	
Stone size, (mm) median [IQR]	10 [10, 12]	10 [10, 12.3]	0.235
Stone density,(HU) median [IQR]	800 [548.3, 1,100]	835 [691, 1,138.5]	0.161
**Hydronephrosis, *n* (%)**			0.107
Grade 1–2	150 (75.7)	35 (64.8)	
Grade 3–4	48 (24.3)	19 (35.2)	

IQR, interquartile range; Pre-op-fever preoperative fever; UAS, ureteral access sheath; WBC, white blood cell; RBC, red blood cell; HB, hemoglobin; NLR, neutrophil-to-lymphocyte ratio; PLR, platelet-to-lymphocyte ratio; LMR, lymphocyte-to-monocyte ratio; SCr, serum creatinine; A/G, albumin-to-globulin; SD, standard deviation; HU, Hounsfield Units. *Statistically significant.

**FIGURE 7:**
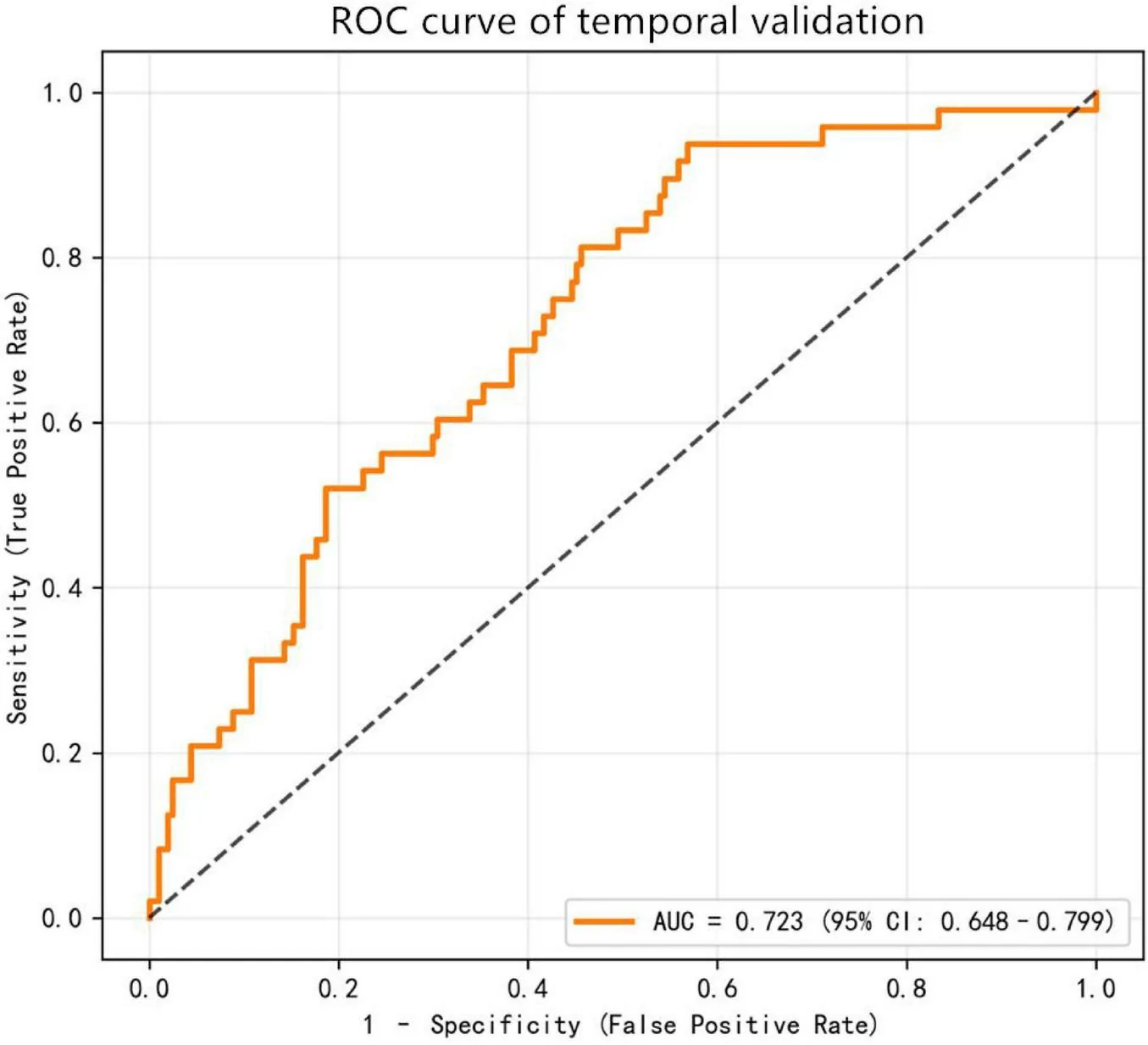
ROC curve of temporal validation (LightGBM).

The receiver operating characteristic (ROC) curve for the temporal validation cohort is displayed in Figure 7; the model attained an area under the ROC curve (AUC) of 0.723 (95% CI: 0.648–0.799), demonstrating acceptable discriminative power to distinguish patients with the target endpoint from unaffected individuals in unseen, temporally independent samples.

The optimal cut-off value of 0.4082 was determined by maximizing the Youden’s J index within the training subset of the derivation cohort, and this predefined threshold was consistently applied to the temporal validation cohort without re-optimization. Model performance metrics in the temporal validation cohort are listed in Table 5. At this optimal threshold, the LightGBM model achieved an overall accuracy of 0.845, accompanied by a sensitivity of 0.601 and a high specificity of 0.912. The F1-score, which balances precision and sensitivity, was 0.625, and the Youden’s J index was 0.513. The statistically high specificity highlights the model’s capacity to minimize false-positive misclassifications among negative cases, whereas the moderate sensitivity indicates a non-negligible risk of false-negative underdiagnosis for positive patients.

**TABLE 5:** Model performance metrics in the temporal validation cohort.

Model	AUC (95% CI)	Accuracy	Sensitivity	Specificity	F1-score	Youden’s J
LightGBM	0.723 (0.648–0.799)	0.845	0.601	0.912	0.625	0.513

Supplementary Figure 2 displays the calibration plot and decision curve analysis (DCA) of the LightGBM model in the temporal validation cohort. In the calibration plot (Supplementary Figure 2A), LightGBM predicted probabilities closely matched observed event rates across all risk thresholds with slight departures from the ideal calibration line, showing satisfactory model calibration. The Brier score was 0.1321, reflecting low overall prediction error and acceptable calibration. Decision curve analysis (Supplementary Figure 2B) revealed clear net clinical benefit of the model. The LightGBM curve outperformed the “treat all” and “treat none” strategies over the threshold range of 0–0.6; net benefit diminished and turned negative above 0.6, meaning the model offers valuable guidance for stratifying patients at mild-to-moderate risk of postoperative fever.

Taken together, these temporal validation findings confirm that the LightGBM model maintains moderate discriminatory performance and moderate temporal generalizability across independent patient populations.

## Discussion

Although fURL exhibits a favorable safety profile, fever (7% to 28%) and sepsis (0.5% to 11.1%) remain the most prevalent complications despite technological advances (21–23). Patients who develop urosepsis or septic shock carry a high mortality risk; even with rigorous treatment, the in-hospital mortality ranges from 2% to 9% (24). Early identification of predictive markers and construction of a perioperative nomogram are critical for predicting postoperative fever after fURL and its progression to urosepsis. Based on large-scale retrospective data, this study adopted multiple machine learning models to achieve early prediction of POF and screen the optimal combination of diagnostic indicators. This work aims to develop more precise predictive tools for clinical practice and provide novel insights for personalized treatment and perioperative risk assessment.

With the increasing application of machine learning algorithms in clinical medicine, several researchers have attempted to construct machine learning-based models for predicting infective complications after fURL. Ekşi et al. (25) established three predictive models for postoperative infectious complications in patients who underwent fURL, utilizing distinct machine learning algorithms. Among these models, the RF model exhibited the most favorable performance, achieving an AUC of 0.956. Additionally, the sensitivity and specificity of the RF model were determined to be 87% and 92%, respectively. Castellani et al. (26) also employed three machine learning algorithms to construct predictive models for sepsis and septic shock after fURL using a cohort of 1,552 patients. The Random Forest model yielded the optimal predictive performance, with a precision of 1.00, a recall of 0.86, an F1 score of 0.92, and an accuracy of 0.92. Li et al. (27) adopted five machine learning algorithms to develop predictive models for postoperative infectious complications following fURL based on a cohort of 833 patients. The eXtreme Gradient Boosting model achieved the optimal predictive performance, with an AUC of 0.858, a sensitivity of 0.877, a specificity of 0.981, and an accuracy of 0.841. In this study, six distinct ML models were employed for the prediction of POF after fURL. The LightGBM model had the strongest discriminative ability, with the highest AUC (0.896 95% CI: 0.853–0.94) and PR-AUC (0.787), and also achieved the highest specificity (0.967) and accuracy (0.891), showing high prediction accuracy. LightGBM is a high-efficiency gradient boosting algorithm. It performs well in analyzing nonlinear, high-dimensional and sparse data. It saves computing resources and runs much faster during model training. Built-in regularization can prevent overfitting and simplify model structure. Meanwhile, it adapts well to large datasets and supports fast parallel calculation. In addition, LightGBM can effectively deal with incomplete and heterogeneous clinical data, lower prediction errors, and show good applicability in clinical predictive research (28).

SHAP is a powerful interpretability approach for interpreting machine learning model predictions. Originating from the game theory concept of Shapley values, it can quantify the marginal contribution of each feature to individual prediction results (16, 29). In this study, SHAP was adopted to quantitatively assess the contribution of each variable to model predictions. SHAP values enable both global and local interpretability of feature effects. The results revealed that midstream urine culture, preoperative fever, and urine leukocyte esterase were the dominant predictors of POF risk, with their average SHAP values statistically higher than those of other variables. In line with the findings of the majority of existing literature (8–10, 30), preoperative UTI serves as a significant risk factor for fever following fURL. For instance, Southern et al. (31) identified preoperative UTI as one of the most significant predictors of POF or systemic inflammatory response syndrome after ureteroscopy. Kayar et al. (32) constructed an interpretable model with six routine perioperative factors for precise prediction of post-fURL sepsis risk, and their favorable predictive performance was similarly credible.

Consistent with the above results, intraoperative UAS use showed a protective statistical association with a lower risk of POF after fURL in this retrospective cohort. The emergence of suction ureteral access sheath (S-UAS) in recent years has highlighted its unique merits in maintaining low pelvic pressure and reducing postoperative fever risk. Unlike the conventional ureteral access sheath, which is typically positioned at the ureteropelvic junction, the tip-bendable suction ureteral access sheath can be advanced into the renal pelvis as needed and directed into specific calices, ensuring adequate space for irrigation-suction maneuvers. This adjustable design helps prevent obstruction of the sheath opening by the ureteropelvic junction mucosa while maintaining low intra-pelvic pressure. When combined with a negative-pressure device, the suction-UAS enables a continuous irrigation-suction cycle, which not only improves intrarenal fluid drainage but also sustains a low-pressure environment (33, 34). An international, multicenter, randomized study by Zhu et al. (4) found that the use of a tip-bendable suction ureteral access sheath resulted in a significantly higher initial stone-free rate compared to the traditional ureteral access sheath group; the incidence of postoperative fever was also notably lower in the S-UAS group than in the T-UAS group (5.6% vs. 17.5%). Qian et al. (5) similarly reported that the use of a suction-UAS during fURL was associated with a higher stone-free rate and a lower incidence of POF or systemic inflammatory response syndrome.

In our machine learning models, midstream urine culture, preoperative fever, urine leukocyte esterase and other laboratory markers were identified as the key predictors of POF following fURL in the overall cohort. Meanwhile, we also detected intraoperative UAS use was negatively correlated with postoperative fever risk in model prediction results, presenting a protective statistical association in this retrospective cohort. Targeted interventions based on these modifiable biomarkers are expected to effectively lower POF risk in high-risk populations. Accordingly, our findings carry modest practical implications for POF prevention and provide preliminary references for perioperative risk stratification and individualized monitoring to reduce postoperative POF risk. Furthermore, SHAP values can generate feature importance rankings at the individual patient level. This enables clinicians to implement targeted interventions for individuals at high risk of POF, instead of adopting a universal intervention strategy for all patients. Such individualized management facilitates more efficient allocation of medical resources, as clinical measures can be tailored to the specific contributing factors of each patient.

Despite the aforementioned strengths, several limitations of the present study need to be acknowledged. First, this study excluded patients aged over 70 years because they usually carry a heavy burden of comorbidities including chronic renal insufficiency, long-term immunosuppression and recurrent urinary tract infections, which may confound independent risk evaluation of postoperative fever. Therefore, the predictive performance of our LightGBM model for elderly patients older than 70 remains unvalidated, and extra external data are required to confirm its applicability in this age group. And its retrospective design introduces the potential for selection bias, which is compounded by the limited sample size. Second, as a single-center study with a relatively small number of participants, only temporal validation was conducted in this study, which may limit the generalizability and widespread application of our predictive model. In addition, the model merely predicts postoperative fever rather than progression to sepsis or septic shock. Multicenter prospective external validation is needed prior to broad clinical adoption. Furthermore, accumulating evidence has confirmed that prolonged operative time and elevated intraoperative intrarenal pelvic pressure are critical risk factors for post-operative fever. Regrettably, these two variables were not included in our predictive model. On one hand, real-time continuous pelvic pressure monitoring was not a routine standardized procedure at our institution during the cohort recruitment period, and complete pressure records could not be retrospectively extracted from surgical documents. On the other hand, operative duration was not mandatory in our hospital’s electronic medical system during the 2019–2024 cohort period; only incomplete unstructured surgical notes contained fragmentary time records without unified digitization, leading to > 25% missing rate for this variable. Exclusion of operative time is a key data-driven limitation rather than deliberate methodological choice, and this may attenuate model predictive performance. *Post-hoc* addition of this variable would lead to data dredging and overfitting, which would impair the statistical reliability of our machine learning model. Finally, while the AUC values of our machine learning models were acceptable, the overall performance can still be improved by adopting and exploring novel algorithms.

## Conclusion

In conclusion, this study developed and validated an interpretable LightGBM model for predicting POF after fURL. The model achieved acceptable predictive performance, with preoperative infection markers and UAS use as key predictors. The SHAP-based interpretability enhances clinical applicability. This model provides a valuable tool for perioperative risk stratification and personalized postoperative monitoring, potentially reducing infectious complications and improving patient outcomes.

## Data Availability

The original contributions presented in this study are included in this article/Supplementary material, further inquiries can be directed to the corresponding authors.

## Footnotes

1
https://www.r-project.org/

2
https://www.python.org/
