# Supplementary material for: Development and validation of an interpretable machine learning model for predicting postoperative fever after flexible ureteroscopic lithotripsy: a single-center retrospective cohort study
Source: Front Med (Lausanne). 2026 Sep 3;13:1913792. doi: 10.3389/fmed.2026.1913792 (PMC13581583; doi:10.3389/fmed.2026.1913792)
Supplement: Supplementary file 1 [file Supplementary_file_1.docx]

Supplemented figures

Supplemented figure S1: Calibration analyses and Decision Curve Analysis in internal test set.(A) Calibration plot. Black dashed line: perfect calibration; orange curve: LightGBM model. (B) Decision curve analysis. Solid black line: treat-none strategy; grey dashed line: treat-all strategy; orange curve: LightGBM predictive model.

Supplemented figure S2: Calibration analyses and Decision Curve Analysis in temporal validation cohort. (A) Calibration curve: dashed line = perfect calibration, orange curve = LightGBM model. (B) DCA: black line = treat none, grey dashed line = treat all, orange curve = LightGBM model.
