# Supplementary material for: Development and validation of an interpretable machine learning model for predicting postoperative fever after flexible ureteroscopic lithotripsy: a single-center retrospective cohort study
Source: Front Med (Lausanne). 2026 Sep 3;13:1913792. doi: 10.3389/fmed.2026.1913792 (PMC13581583; doi:10.3389/fmed.2026.1913792)
Supplement: Supplementary file 2 [file Table_1.docx]

Supplementary Table S1 the baseline characteristics of patients in the derivation and temporal validation cohorts.

|  | derivation cohort | temporal validation cohort | P value* |
| --- | --- | --- | --- |
| Age, years median ± SD | 51.31±10.26 | 52.07±12.81 | 0.774 |
| Sex, n (%) |  |  | 0.239 |
| Female | 347 (34.5) | 97 (38.4) |  |
| Male | 658 (65.5) | 155 (61.6) |  |
| Hypertension, n (%) |  |  |  |
| No | 667 (66.3) | 165 (65.4) | 0.789 |
| Yes | 338 (33.7) | 87 (34.6) |  |
| Diabetes, n (%) |  |  | 0.495 |
| No | 889 (88.4) | 219 (86.9) |  |
| Yes | 116 (11.6) | 33 (13.1) |  |
| Pre-op-fever, n (%) |  |  | 0.554 |
| No | 888 (88.3) | 226 (89.6) |  |
| Yes | 117 (11.7) | 26 (10.4) |  |
| Post-op-fever, n (%) |  |  | 0.880 |
| No | 794 (79) | 198 (78.5) |  |
| Yes | 211 (21) | 54 (21.5) |  |
| UAS, n (%) |  |  | 0.765 |
| No | 212(21.1) | 51(20.2) |  |
| Yes | 793(78.9) | 201(79.8) |  |
| Pre-stented, n (%) |  |  | 0.557 |
| No | 810(80.5) | 207(82.1) |  |
| Yes | 195(19.5) | 45(17.9) |  |
| Urine.WBC, (/μL) median [IQR] | 36.5 [12,892.5] | 34[8,265] | 0.531 |
| Urine.leukocyte.esterase, n (%) |  |  | <0.001 |
| 0 | 631(60.9) | 192(76.1) |  |
| 1+ | 128(12.7) | 43(17.1) |  |
| 2+ | 94(9.3) | 9(3.5) |  |
| 3+ | 152(17.1) | 8(3.3) |  |
| Urine.nitrite, n (%) |  |  | 0.410 |
| No | 939(93.4) | 239(94.8) |  |
| Yes | 66(6.6) | 13(5.2) |  |
| Mid.urine.culture, n (%) |  |  | 0.516 |
| No | 795(79.1) | 204(80.9) |  |
| Yes | 210(20.9) | 48(19.1) |  |
| WBC, (×10^^9^/L) median [IQR] | 6.62 [5.2,8.5] | 6.51 [6,11.7] | 0.754 |
| RBC, (×10^^12^/L) mean ± SD | 4.43±0.4 | 4.58±0.5 | 0.621 |
| Platelet, (×10^^9^/L) mean ± SD | 225.02±61.36 | 228.69±66.72 | 0.428 |
| HB, (g/L) mean ± SD | 138.6±16.4 | 135.3±14.1 | 0.247 |
| NLR, median [IQR] | 2.73 [1.61, 4.21] | 2.81 [1.9,5.6] | 0.582 |
| PLR, median [IQR] | 164.44 ± 26.2 | 176.48 ± 29.4 | 0.463 |
| LMR, median [IQR] | 3.43 [2.33, 4.47] | 3.16 [2.15, 4.63] | 0.539 |
| SCr, (μmol/L) median [IQR] | 78.1 [64.2,98.1] | 78.2 [59.4,106.5] | 0.883 |
| Total.bilirubin, μmol/L median [IQR] | 15.1[11.2,19.9] | 14.9 [11.3,20.4] | 0.341 |
| A/G, median [IQR] | 1.6[1.4,1.8] | 1.7[1.5-1.8] | 0.544 |
| Stone side, n (%) |  |  | 0.001 |
| Left | 529 (52.6) | 102 (40.4) |  |
| Right | 476 (47.4) | 150 (59.6) |  |
| Multiple.stones, n (%) |  |  | 0.572 |
| No | 590 (58.7) | 143 (56.7) |  |
| Yes | 415 (41.3) | 109 (43.3) |  |
| Stone size, (mm) median [IQR] | 10 [10,12.7] | 10 [10,12.3] | 0.884 |
| Stone density,(HU) median [IQR] | 812 [589, 1089] | 816 [556.2,1113.5] | 0.652 |
| Hydronephrosis, n (%) |  |  | 0.709 |
| Grade1-2 | 726 (72.2) | 185 (73.4) |  |
| Grade3-4 | 279 (27.8) | 67 (26.6) |  |

IQR interquartile range; Pre-op-fever preoperative fever; UAS ureteral access sheath; WBC white blood cell; RBC red blood cell; HB hemoglobin; NLR neutrophil-to-lymphocyte ratio;PLR platelet-to-lymphocyte ratio; LMR lymphocyte-to-monocyte ratio; SCr serum creatinine;A/G albumin-to-globulin; SD Standard Deviation; HU Hounsfield Units.

* Statistically significant.
